# Definition of professionalism and tools for assessing professionalism in pharmacy practice: a systematic review

**DOI:** 10.3352/jeehp.2019.16.22

**Published:** 2019-08-21

**Authors:** Huda Dubbai, Barbara-Ann Adelstein, Silas Taylor, Boaz Shulruf

**Affiliations:** 1 Office of Medical Education, Faculty of Medicine, The University of New South Wales, Sydney, Australia; 2 Prince of Wales Clinical School, Faculty of Medicine, The University of New South Wales, Sydney, Australia; 3 Centre for Medical and Health Sciences Education, Faculty of Medical and Health Sciences, University of Auckland, Auckland, New Zealand; Hallym University, Korea

**Keywords:** Professionalism, Pharmacists, Community Pharmacy Services

## Abstract

**Purpose:**

In contemporary pharmacy, the role of pharmacists has become more multifaceted, as they now handle a wider range of tasks and take more responsibility for providing patient care than 20 years ago. This evolution in pharmacists’ responsibilities has been accompanied by the need for pharmacists to display high-quality patient-centred care and counselling, and to demonstrate professionalism, which now needs to be taught and assessed as part of pharmacy education and practice. This study aimed at identifying definitions of professionalism in pharmacy practice and critically evaluating published instruments for assessing professionalism in pharmacy practice.

**Methods:**

We searched the medical literature listed in Scopus, MEDLINE, and PsycINFO databases from 1 January 2000 to 31 December 2018. All papers meeting our selection criteria were reviewed and summarised into a clear review of professionalism requirements in pharmacy practice. Details of the instruments measuring professionalism were reviewed in detail.

**Results:**

There is no accepted simple definition of professionalism, although we identified several theoretical and policy frameworks required for professional pharmaceutical practice. We identified 4 instruments (the Behavioural Professionalism Assessment Instrument, Lerkiatbundit’s instrument, the Pharmacy Professionalism Instrument, and the Professionalism Assessment Tool that build on these frameworks and measure professional practice in pharmacy students. These were found to be reliable and valid, but had only been used and tested in student populations.

**Conclusion:**

Given the increasingly broad role of community pharmacists, there is a need for assessments of professionalism in practice. Professionalism is a complex concept that is challenging to measure because it has no standardised definition and the existing literature related to the topic is limited. Currently available instruments focus on measuring the development of the elements of professionalism among pharmacy students, rather than pharmacists.

## Introduction

### Rationale

Community pharmacy accounts for one of the largest groups of healthcare providers in the world [1], and constitutes an important and expanding healthcare resource for communities in terms of their access to medicines and to health and pharmaceutical advice [2]. The scope of practice of pharmacy has changed substantially over the last 20 years. Traditionally, pharmacists were informal advisers on monitoring medication usage and might have provided some counselling on correct drug information; however, a notable change in recent decades in the pharmacy profession has been the expansion of the role of pharmacists, allowing them to have the authority and accountability needed to achieve complete autonomy in their career. This means that a pharmacist can manage many medication decisions without referral to the prescriber [3]. Pharmacy practice is now focused on pharmaceutical care, rather than on the soft knowledge and skills that were emphasised previously, such as preparing and supplying medicines in patient care [4,5]. Accordingly, the role of the community pharmacist has shifted significantly towards providing more patient-centred care and counselling [1,4,5]. The current health regulatory bodies in Australia, such as the Australian Health Practitioner Regulation Agency, emphasise the importance of the pharmacist’s clinical role in promoting advanced pharmaceutical care practices and applying the highest possible level of professional competence [6,7].

### Objectives

This study aimed at identifying the definition of professionalism, particularly in relation to pharmacy practice, and critically appraising published instruments for assessing professionalism in pharmacy practice. It sought to identify gaps in the literature with a view to improving professionalism and education of pharmacists—and hence, care provision—in pharmaceutical practice.

## Methods

The review was conducted in accordance with the Preferred Reporting Items for Systematic Reviews and Meta-Analyses guidelines [8].

### Information sources

This scoping literature review comprised studies identified by searching the Scopus, MEDLINE, and PsycINFO electronic databases. The scoping approach is flexible, as it is not limited to a specific method and strategy and aims to identify a wide range of relevant studies with the goal of determining whether further research is required to explore the topic [9].

The purpose of using scoping in this study was to map the current literature on a specific theme to identify the main concepts underpinning the subject and to investigate existing research gaps to inform pharmacy practice. To do this, the search keywords and terms used were combinations of different derivations of the following words: professionalism, pharmacy, measure, assess, instrument, and tool. Reference lists from those papers identified as pertinent were also examined, and any paper deemed relevant was also considered. Definitions, models, and assessment of professionalism in pharmacy were explored through all papers from the sources.

### Eligibility criteria for professionalism measurements

All papers that assessed professionalism elements such as communication and interaction, estimated students’ opinions regarding any professionalism domain such as accountability and autonomy, or used different evaluation forms to introduce students to professional values and enhance patient-centred professionalism in pharmacy were included. Only papers published from 1 January 2000 to 31 December 2018 were included. Studies not written in English, and those that were generally descriptive only or based solely on opinion, were excluded.

### Search

The search strategy consisted of the following:

(Professionalism) → (“Professionalism.mp” in MEDLINE and PsycINFO; “TITLE-ABS-KEY (professionalism )” in Scopus)
AND
(pharmac*) → (“pharmac*.mp” in MEDLINE and PsycINFO; TITLE-ABS-KEY ( pharmac* ) in Scopus)
AND
(assess* or test* or instrument* or measur* or scale* or tool* or evaluat*) → (“assess*….mp” in MEDLINE and PsycINFO; ALL ( assess*… ) in Scopus)

### Study selection

**Fig. 1.**
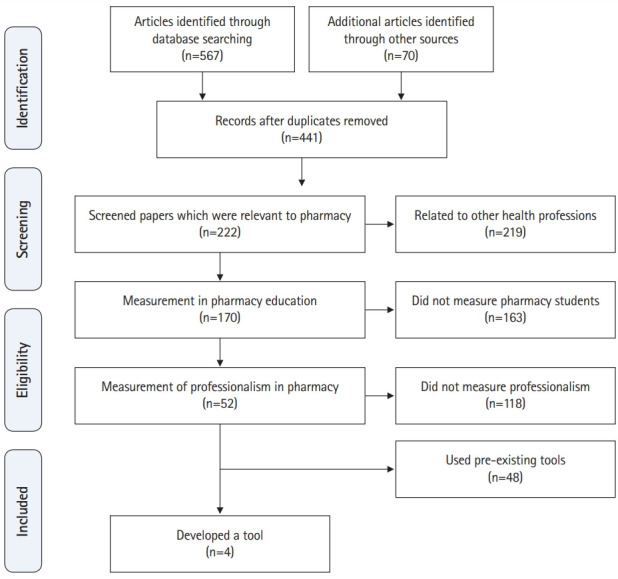
The strategy of the scoping review.

The search yielded 567 studies, of which 52 related particularly to professionalism in pharmacy (Fig. 1). Of these, only 4 studies dealt with the development or use of an instrument or tool to measure professionalism in pharmacy practice. These 4 instruments focused on the effect of curricula in enhancing professionalism among students or assessing pharmacy students by exploring their perceptions of aspects of professionalism.

### Data collection process, data extraction and sample size

The papers were selected by the author (HD) according to the eligibility criteria. The 4 eligible papers were further examined in detail by all authors. Author, year of publication, participant type, sample size, factors, reliability (Cronbach’s α), and type of validity were recorded for each eligible paper. In scale development papers, there is no risk of bias to be reported. However, we were concerned about whether the sample size was adequate. The sample size is recommended to be larger than 300, but a size of 150 is sufficient [10].

### Data items

As described above for data collection, author, year of publication, participant type, sample size, factors, reliability (Cronbach’s α), and type of validity were recorded for each eligible paper.

### Sample sizes of studies

As discussed above, in scale development papers, there is no risk of bias to be reported. However, we were concerned about whether the sample size was adequate. The sample size is recommended to be larger than 300, but a size of 150 is sufficient.

### Summary measures

None (not applicable in scale development papers).

### Synthesis of results

None (not applicable in scale development papers).

### Risk of bias across studies

None (not applicable in scale development papers).

### Additional analyses

None (not applicable in scale development papers).

## Results

### Study characteristics

This topic is addressed in the “Professionalism measurements in the literature: study characteristics” section in the paragraphs below.

### Sample sizes of studies

This topic is addressed in greater detail in the “Professionalism measurements in the literature: study characteristics” section below. In all included papers, the number of study participants was between 231 and 1,202, which suggested sufficient sample sizes for scale development.

### Results of individual studies and synthesis of results

The results of individual studies are discussed in the paragraphs below; synthesis of results is not applicable to scale development papers.

### Definitions of professionalism

While professionalism is recognised as an important quality by professional bodies and higher education providers [11,12], its definition may not be sufficiently clear to practitioners [11], which does not facilitate accurate measurements [13,14]. Definitions and measurements of professionalism in the pharmacy profession have been widely debated in the literature, and finding a holistic definition of professionalism with general acceptance is difficult. Existing definitions focus on, or are built on, descriptive lists of professional tasks. In 1999 the American Pharmacists Association, Academy of Students Pharmacists (APhA–ASP) and the American Association of College of Pharmacy (AACP) Task Force on Pharmacy Professionalism outlined the following 10 essential domains that professional pharmacists should demonstrate in their practice: knowledge and skills of the health profession; service orientation; creativity, innovation and initiative; effective relationships with others; conscience and honesty; commitment to self-improvement through lifelong-learning; ethically sound decision making; leadership; pride in profession; and accountability [13,15].

The Task Force then developed a white paper to promote and evaluate professionalism within pharmacy education programmes [14]. The white paper defined professionalism as “active demonstration of the traits of a professional … displaying values, beliefs, and attitudes that put the needs of another above your personal needs” [16,17].

In addition to the 10 domains of professionalism, the AACP Task Force added an 11th element: punctuality and flexibility [14,18], on the basis that being punctual and flexible is an essential reflection of one’s professional reliability and career values [19].

Professionalism has also been considered in other health care professions. In medicine, for example, the American Board of Internal Medicine (ABIM) outlined 3 commitments to professionalism for physicians: a commitment to sustain the interest and well-being of patients; accountability to quality excellence; and a commitment to be responsive to the health requirements of the community. These were followed by 6 additional domains of professionalism expected of physicians: (1) excellence in performance to meet or exceed ordinary expectations and the pursuit of lifelong learning and individual development; (2) altruism in the concern for and prioritising the welfare of patients above self-interest; (3) accountability in being responsible for responding to patients as well as for society and the health profession; (4) honour and integrity, being fair and honest and reflecting credibility in performance; (5) duty of care, including the commitment to assure the safety and health of the patient through provision of high-quality patient care; and (6) respect for others, including patients, their families, peers at work, and other healthcare professionals [20].

Chalmers’ definition of professionalism, which calls for displaying consideration and showing respect for others, empathy, commitment, maintaining boundaries, and confidentiality, harmonises with the definition of the ABIM [16,21]. Hammer et al. [16] further modified the 6 domains of professionalism proposed by the ABIM to suit pharmacy students, particularly noting responsibility, initiative, maturity, appearance, competence, standards, and interpersonal communication skills.

According to the American College of Clinical Pharmacy, to demonstrate professionalism, practicing pharmacists must obtain knowledge during their studies and, following graduation, develop the professional attitudes and behaviours necessary to deliver quality pharmaceutical care [19]. In 2014, the International Pharmaceutical Federation defined professionalism for the pharmacist as complying with the quality of behaviours and respect guided by attitudes and moral values, with an additional commitment to achieve what is expected of practitioners to uphold public trust in the profession [22].

The term ‘professionalism’ has been used in different ways. Professionalism has been defined as exhibiting beliefs, principles, and attitudes that serve the best interests of patients above practitioners’ personal interests [23]. Professionalism, in general, can be defined as being useful to the community, acquiring autonomy to allow the individual to make professional judgments without outside help from others, having a sense of responsibility, and performing one’s duty regardless of external rewards. Providing a definition of pharmacy professionalism involves, therefore, a complete clarification of the distinct role of pharmacists, rather than just providing a list of professional components [11]. Professionalism can also be described as behaviour or value aims that distinguish a profession [13].

Debated concepts and interpretations have evolved over time [24] and, while the definitions discussed above grasp the core of professionalism and share many of the elements of professional practice, the actual elements are mostly descriptive, relatively wide-ranging, and imprecise. As the American College of Clinical Pharmacy et al. [25] point out, “no single definition can encompass all applications”; each application is a combination of specific competences, beliefs, and behaviours. Brown and Ferril [26] highlighted that the existing definitions of professionalism in pharmacy focus on devising lists of behavioural elements and do not touch on the depth of interactions that occur within the professional relationship, such as dealing with patients and the healthcare team [27]. The opinions of Brown and Ferril [26] align with Kelley et al. [28], who stated that building on previous definitions without clarification can lead to seeing definitions of professionalism as a set of attitudes and behaviours [28]. There is a need, therefore, to develop a clear, measurable definition of professionalism that encompasses all the required elements in health professions [29].

### Professional behaviours and attitudes

It is clear that some of the previous definitions describe professionalism as a set of behaviours and attitudes, in addition to specific competencies such as knowledge. Professionalism can also be constructed as a set of internally held principles that can be measured through behaviours [28]. Future pharmacists should acquire these critical elements as part of their professional pharmacy education [19], and be encouraged to reflect on them in their daily activities after they graduate. While professionalism can be recognised when it exists, specifying its exact components is more difficult [23]. With this in mind, an explanation of behaviour and attitude and the relationship between them, in the context of pharmacy professionalism, is an essential element of professional education [13,30]. Additional clarification of the dimensions of behaviours and attitudes will help practitioners determine which elements must be established and measured [13]. As Hammer [17] points out: “Professionalism can be defined by the way it is demonstrated in practice, by its structural items, by the views held by those in the profession or in a values-based manner.” This suggests that professionalism is often exhibited by the demeanour of the pharmacist in professional situations. Thus, professional behaviours relate to how practitioners conduct themselves [16]. Professional behaviour may also reflect a person’s internal moral compass. Such internal attitudes, however, are based on a set of internal principles and beliefs that may not be fully aligned with actual practices [28]. This aspect makes measurement difficult because only what is observable can be measured; external behaviour is observable, but underlying internal attitudes are not.

Nonetheless, many have attempted to measure attitudes as a predictor of behaviour [31,32]. The pharmacist may, however, strive to exhibit professional behaviour, yet simultaneously hold ‘unprofessional’ attitudes [13,33]. This leads to the question: what is the benefit of learning professional attitudes and scientific knowledge without demonstrating suitable behaviour and performance? For example, is it professional for a pharmacist to give a patient health information, but not to assist the patient in making a decision about managing his or her health?

The following scenario illustrates the difficulty of measuring attitudes versus behaviours in pharmacy and shows how difficult it is to measure a pharmacist’s intentions or professional attitudes compared to measuring his or her actual performance and behaviours. As a hypothetical example, a pharmacist gives a patient drug information regarding a new medicine which, although it is more effective than the old medication, has some unpleasant side effects. The patient finds it difficult to choose whether to continue taking the old medication or to try the new one. A pharmacist with good internal intention (professional attitude) might believe that his or her duty is to provide good information and answer any questions the patient may ask, but ultimately believes the decision of which medication to take is entirely the patient’s personal responsibility.

A pharmacist demonstrating professional behaviour, on the other hand, will encourage the patient to ask questions, address those questions and concerns, and suggest solutions to any problems identified by the patient. He or she will do as much as possible to assist the patient to come to the best decision. Thus, the internal intention (attitude) of the pharmacist to assist the patient only if he or she is asked to do so is not enough to demonstrate professionalism, because no assisting behaviour is being carried out by the pharmacist.

This example demonstrates how behaviours can be easily measured, but the underlying attitudes of the professional are not easily determined. It must be noted, however, that since attitudes and behaviours are not independent of each other, practising particular professional behaviours may also, over time, change the practitioner’s professional attitudes [7].

### Models of professionalism in pharmacy

**Fig. 2.**
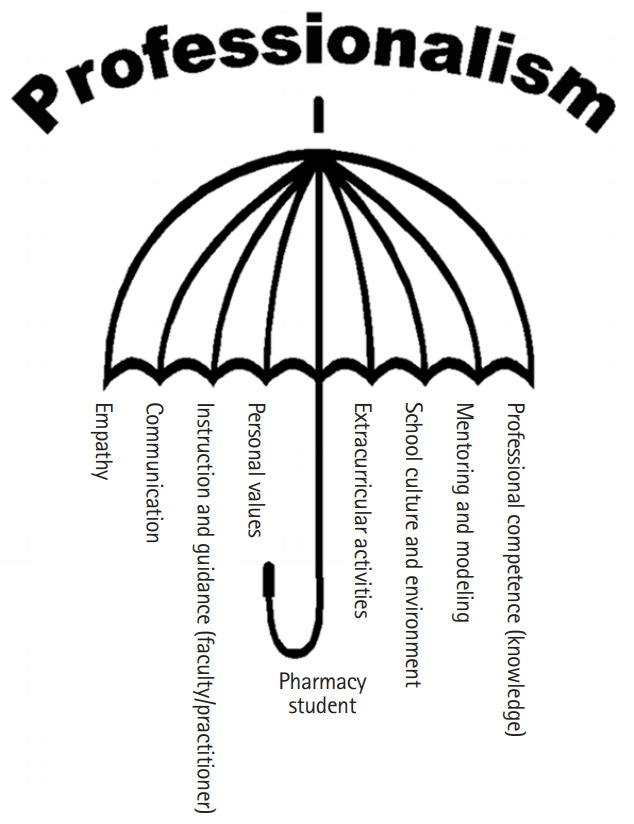
The umbrella model of professionalism.

Researchers have conceptualised various models to describe the range of elements needed to be a professional pharmacist, including the umbrella model [13] and the bicycle wheel model [25]. In the umbrella model, the umbrella represents the holistic role of pharmacy education in fostering a student’s future professionalism and places the student (the future pharmacist) as the umbrella’s centre shaft. The ribs radiating from the central shaft represent professionalism domains such as communication skills, empathy and competence, personal values, and knowledge (Fig. 2) [13].

Some of the duties of pharmacists include protecting patients’ confidentiality and preventing harm caused by medications. Similarly, the umbrella model of professionalism serves as a symbol of how pharmacists act to protect patients’ safety and maintain the integrity of pharmaceutical practice.

**Fig. 3.**
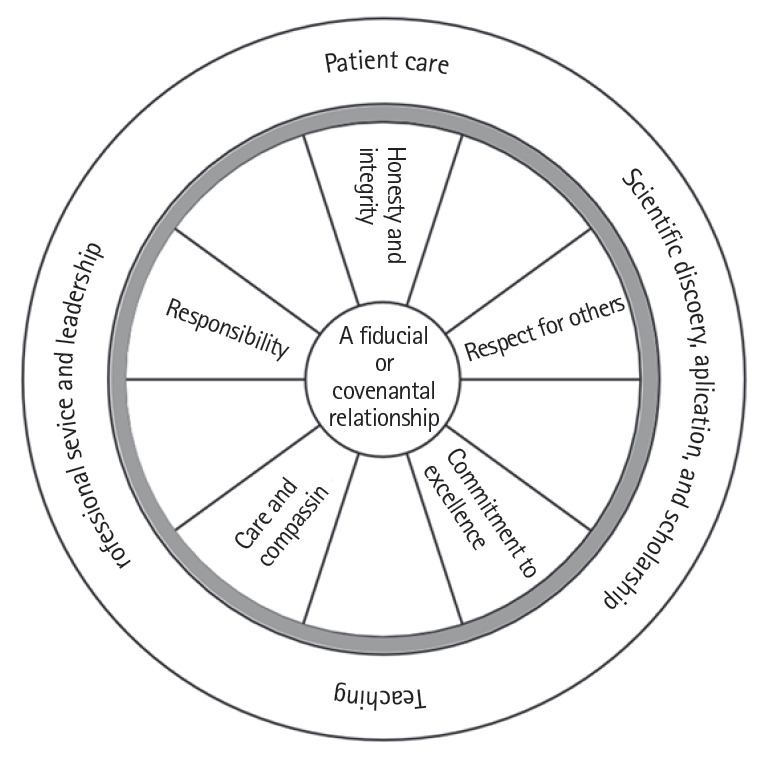
The bicycle wheel model of professionalism.

In the bicycle wheel model, the wheel hub includes a set of critical values, such as integrity and altruism, and the radiating spokes represent pharmacists’ behaviours, such as empathy and respect. The tyre connects all the elements of professionalism and includes aspects such as adhering to professional attire and punctuality. In this model, practitioners’ values are the core of professionalism (Fig. 3) [25] and professionalism for pharmacists relies mostly on performance, interaction, and professional behaviour with patients on a daily basis.

Professionalism is an active, multi-domain concept. Likewise, the bicycle wheel model is dynamic in its regular rotations and spokes. Each spoke signifies a domain of professionalism, which jointly acts to reinforce the wheel. In this model, principles such as honesty and sense of responsibility occupy the centre of the wheel and depict the importance of incorporating such principles into pharmacists' practice, for instance, communication while counselling patients.

Optimal medication management requires active interpersonal collaboration among pharmacists, patients, and healthcare professionals. As pharmacists’ roles change, making them more actively involved in direct patient care, effective communication with patients becomes crucial. Such models may help clarify some of the elements of professionalism and elucidate the relationships between professionalism domains in general and some of the features of the pharmacy profession in particular. These models can facilitate the process of developing and updating professional concepts in pharmacy practice.

### Assessment of professionalism in pharmacy

Maintaining and enhancing professional behaviour requires measurements. A valid measurement is essential for assessing any behaviour [34]. Berenson [35] emphasised the importance of measurement processes, stating that, “if you cannot measure it, you cannot manage it,” meaning that unmeasured parameters cannot be improved [36]. A key feature in the measurement of pharmaceutical professional practice lies in the ability to collect quantified data that can be used in career development, creating a subsequent positive effect on professional performance. The creation of instruments for gathering standardised, measurable data for pharmacists plays a key role in ensuring that the activities of practising pharmacists are suitable, and in evaluating their level of performance to foster their professional growth [37]. Collecting various perspectives from multiple evaluators increases the range of assessment, and in turn the validity and reliability of the measurement [38]. Some studies have examined the development of elements of professional behaviour among students, rather than pharmacists [39,40]. Brown and Ferrill [26] noted, however, that behavioural assessments conducted in pharmacy schools before graduation are prone to be influenced by the students’ enthusiasm to achieve good grades, rather than being reflective of the student’s true values. In contrast, a workplace survey for pharmacists can provide beneficial summative information regarding their professionalism, based on their real performance as practising pharmacists [26]. Collecting such summative data and using feedback is important for evaluating performance, while collecting formative data from learning environments and using feedback is important for improving curricula. Therefore, there is a need to find an effective mechanism or set of instruments for measuring the professionalism of practising pharmacists.

### Professionalism measurements in the literature: study characteristics

This scoping literature review identified 4 important instruments that have been developed to measure professionalism among pharmacy students and 2 examples of how particular instruments have been applied in pharmacy settings.

The 4 instruments identified from the literature review are: (1) Behavioural Professionalism Assessment Instrument (BPAI) [16]; (2) Lerkiatbundit’s instrument [41]; (3) Pharmacy Professionalism Instrument (PPI) [42]; and (4) Professionalism Assessment Tool (PAT) [28].

All 4 instruments involved developing and refining a survey to improve validity and consistency. Of these, 2 measured professional behaviours among students [16,28], and 2 studies explored the measurement of professionalism attitudes [41,42]. Chronologically, Hammer et al. [16] created an objective survey tool for measuring professional behaviours in pharmacy, based on various student evaluation forms collected from pharmacy and medical schools. Lerkiatbundit [41] reviewed a previously published instrument and refined and adapted it to measure professional attitudes. Chisholm et al. [42] based their survey items on measuring the professional attitudes detailed in the 6 domains of professionalism proposed by the ABIM among student populations; and finally, in 2011, Kelley et al. [28] developed a survey instrument to measure professional behaviours in pharmacy education. In all included papers, the number of study participants was between 231 and 1,202, which suggested sufficient sample sizes for scale development.

### Results of individual studies

#### Behavioural Professionalism Assessment Instrument

Hammer et al. [16] developed this instrument to measure professional behavioural aspects of pharmacy students’ competence during postgraduate training. They developed a 25-item instrument called the BPAI [12] and applied it to 994 student-and-teacher pairs at 17 schools of pharmacy. Exploratory factor analysis identified 4 factors: responsibility, interpersonal relations/social skills, communication skills, and appearance. Although Hammer et al. [16] did not include in their sample students from different academic years of pharmacy, the scale was later used in a number of other studies [7,26,40]. Each factor yielded high Cronbach α coefficients: 0.947, 0.949, 0.877, and 0.829, respectively, which confirmed the factors’ reliability. The overall reliability of the tool was found to be high (Cronbach α=0.972). The very high values for Cronbach α could be explained by strong interrelatedness between items, or possibly because the items asked the same question, but in a different way [43].

The factor analysis supported the factorial validity of the questionnaire, suggesting that items in the survey successfully assessed the themes intended to be measured. The inter-scale correlation coefficient generated showed that responsibility was correlated with the other 3 factors (interpersonal relations/social skills, communication skills, and appearance), with coefficients of 0.741, 0.703, and 0.653, respectively. Interpersonal relations/social skills showed correlations with communication skills and appearance, with coefficients of 0.688 and 0.547, respectively. Communication skills and appearance were also correlated, with a coefficient value of 0.500. The high correlations between variables may suggest some dependence between the factors. In addition, the strong positive relationship between variables may be directly related to item reliability.

In the study of Hammer et al. [16], students were invited to evaluate their behaviours, and teachers were invited to rate their student’s professional behaviours based on what they observed during the course. The instrument items were reviewed twice by 90 expert teachers and academics from 49 schools of pharmacy, before and after the pilot study, which supported the content validity of the instrument [16].

Example of the application of the BPAI: Using the BPAI developed by Hammer et al. [16], a recent study by Thurston et al. [12] in 2018 measured and compared students’ views on professional attitudes and behaviour at a single school of pharmacy. Out of 482 students, a total of 362 first-, second-, and third-year pharmacy students participated in the study, providing a response rate of 75%. Participants completed the BPAI survey to assess professional behaviours in classes. The results generally showed a substantial difference in views of professionalism among the first-, second-, and third-year students [12].

#### Lerkiatbundit’s instrument

The second instrument in this review was developed by Lerkiatbundit [41] in 2005, based on earlier work by Schack and Hepler [44]. Lerkiatbundit’s instrument was a survey that measured changes in the professional attitudes of students across different curricula and classes in a single school of pharmacy in Thailand from 1998 to 2004. The survey was repeated each year for 5 years and was administered twice to all students in all academic years [41].

In testing the survey, Lerkiatbundit [41] reported that 486 of 622 students (78.1%) completed the survey. Response rates ranged from 70.0% for second-year students to 89.1% for fifth-year students. The instrument in their study covered 6 factors of professionalism: professional commitment, professional organisation, autonomy, public service, self-regulation, and continuing education. The Cronbach α values of the 6 factors were over 0.70, suggesting acceptable reliability. The factor correlation was 0.29 in the 1998 sample and 0.46 for the sample in 2004, which suggests that each factor had a discrete underlying theme. The results of the study showed that students had similar views during their studies and as graduates regarding their interpretation of professionalism items, suggesting that professional attitudes were relatively stable over time, even as participants gained professional skills. A 6-factor correlated model showed better results for measuring attitudinal professionalism factors than other models, such as a 1-factor model and an uncorrelated factor model. This study provided useful insights into individual students’ development in professionalism across different curricula. However, the developed instrument may only be applicable to the study population. Interpretations and responses to the same questions may differ among participants; for instance, interpretations of the questions may be influenced by previous experiences. Thus, it is vital to specify the similarity of the factor structure in different populations to allow the instrument to be modified before use in other cultural contexts [1,45].

#### Pharmacy Professionalism Instrument

The third instrument study was conducted by Chisholm et al. [42], who measured professionalism among first-year students and new pharmacy graduates during their postgraduate training. The main purpose of this study was to create and validate an instrument to assess the attitudinal features of professionalism in pharmacy.

In their research, Chisholm et al. [42] sought to fill the gap in the self-assessment instrument developed by Hammer et al. [16], which lacked an adequate description of the development of professionalism concepts among pharmacy students from the early years of study through graduation. Noting that such a gap in the instrument description may undermine the construction of the instrument, Chisholm et al. [42] used a focus group to extract and create scale items based on the 6 characteristics of professionalism from the ABIM, namely: excellence, respect for others, altruism, duty, accountability, and honesty/integrity. The resultant 18-item instrument they developed was called the PPI. Of 133 first-year students who were approached, 130 responded and completed the questionnaire (97.7%); and 101 of 125 new graduates responded (80.8%). The results of Chisholm et al. [42] confirmed that both student groups had similar views of professionalism. The correlation range for the 6 factors of professionalism was 0.25–0.57, which indicated a discrete factor structure. The reliability of the overall (18-item) questionnaire was 0.82. The Cronbach α values for items in the questionnaire were as follows: excellence (0.75), respect for others (0.72), altruism (0.83), duty (0.77), accountability (0.83), and honesty/integrity (0.85), suggesting satisfactory reliability of the instrument.

One of the major criticisms of the study of Chisholm et al. [42], however, is its measurement of the students during their first year of study rather than across different academic years [46]. Not obtaining such measurements across different school years potentially misses an opportunity to capture data across different programs. Despite this criticism, the scale of Chisholm et al. [42] has served as the first step towards an instrument to measure students’ professionalism [15]. The developed survey items seem relatively good, as the statistical results showed that PPI is reliable for measuring professionalism in students. Additionally, the results support the instrument’s content validity as a measure of pharmacy students’ professional attitudes, as its development was based on revisions and refinements by a panel of experts. The main limitation is that the instrument was only used for students and did not include practicing pharmacists.

Example of application of the PPI: Acting on their criticism that the study sample of Chisholm et al. [42] did not include different programs, Poirier and Gupchup [46] used the PPI questionnaire to compare professionalism elements scores across first-, second-, and fourth-year students at a single school of pharmacy. In 2009 the survey was distributed 3 times across different classes, twice for students in 2010 and 2011, and finally once among class cohorts in 2012.

Internal consistency was calculated for all classes using Cronbach α values. The Cronbach α for professionalism scale for students in different classes was greater than 0.8, suggesting good internal consistency for each scale. Regarding the subscales, Cronbach α ranges were calculated. For both the respect for others and excellence factors across the different classes, the scores were 0.63–0.75 and 0.66–0.77, respectively. This suggests low, yet acceptable, reliability. Other factors in the professionalism scale including altruism, duty, accountability, and honour/integrity yielded a Cronbach α reliability coefficient below 0.5 in the subscales. A score below 0.70 suggests that not all the items within a particular factor measure the same underlying construct. Tavakol and Dennick [43] suggested that this can be confirmed by constructing a correlation matrix for identifying items that are poorly correlated with other items; it may then be possible to remove these identified items from the analysis. Findings of Poirier and Gupchup [46] showed a significant change in professionalism scores between first-year and fourth-year students in relation to accountability, honour/integrity, and altruism. They concluded that a substantial increase in general professionalism attitudes had occurred between the first-year beginners and fourth-year students who had completed their study, suggesting that studying in pharmacy school may enhance some professionalism concepts among students [46,47].

#### Professionalism Assessment Tool

The fourth important instrument review is that of Kelley et al. [28] who developed and cross-validated an instrument to measure the professionalism behaviours of pharmacy students within curricula. Kelley et al. [28] created an instrument called the PAT, a 5-point instrument using the 5 performance levels described by George Miller [48]. These performance levels are: “knows, knows how, shows, shows how, does, is” [28,48]. The items of their scale mapped to items from 3 previous sources including the APhA–ASP/AACP white paper [49], Hammer et al. [16], Chisholm et al. [42], and Kelly et al. [28]. The developed 33-item PAT included 5 domains: (1) upholding principles of integrity and respect; (2) relationships with others; (3) reliability, responsibility, and accountability; (4) citizenship and professional engagement; and (5) lifelong learning and adaptability. These 5 domains were built upon Miller [50]’s original pyramid of competence describing 4 domains of competence.

This instrument was applied to 1,202 first- and third-year students at 7 schools and colleges of pharmacy in America. All 33 items with a factor loading of more than 0.5 on 1 factor showed good validity of the developed scale. Factor loading expresses the relationship of each item (variable) with the underlying factor. Eight items loaded on the factor ‘upholding principles of integrity and respect,’ with the 2 highest-loaded items being: ‘being respectful of colleagues and patients’ and ‘maintaining honesty and integrity in academic and professional contexts,’ with loadings of 0.87 and 0.86, respectively. In contrast, 9 items loaded on the factor ‘relationships with others.’ The two lowest-loaded items for this factor were: ‘work with team to effect change and resolve conflict’, and ‘providing effective and constructive feedback,’ with loadings of 0.59 and 0.55, respectively.

The Cronbach α ranges for each of the 5 domains were 0.91–0.95, suggesting strong reliability. Furthermore, the findings supported internal validity, since the PAT was piloted across different schools of pharmacy and student populations and the combined data achieved a Cronbach α of 0.77. This result encouraged the team to expand the main study to cover 7 schools and colleges. In this large-scale study, the PAT successfully measured professional behaviours in students. The internal consistency of the statistical results showed strong positive correlations among the factors, which suggested robust reliability. Furthermore, the instrument was developed based on previous studies [16,42,49] and experts’ agreement on the professionalism items was included to provide construct validity.

### Risk of bias across studies

None (not applicable in scale development papers).

### Additional analysis

None (not applicable in scale development papers).

## Discussion

### Summary of evidence

Only 4 instruments measuring professionalism in pharmacy practice were identified in the literature. The 4 instruments were similar in terms of the targeted population, namely, students. Furthermore, the instruments were developed after 2000. All the instruments built directly on the 6 domains of professionalism proposed by the ABIM [16] and the instruments were in accordance with the 2000 white paper on student professionalism published by the American Pharmaceutical Association–Academy of Students of Pharmacy/American Association of Colleges of Pharmacy–Council of Deans Task Force on Professionalism [49]. Additionally, in developing these instruments, the assessment method of professionalism used in the 4 tools focused on self-administered surveys with rating scales.

### Limitation

The included measurements used a self-reporting format. Response bias is a problem in research using self-reporting instruments. This bias is greater for questions relating to the quality of performance of the person self-reporting, with people tending to present their performance as better than it is [51]. An observational approach to assess performance may be better as it less biased, but it is technically complicated and challenging to do, with a need for more resources, and may have its own inherent biases. A possible way to minimise bias in self-reporting is to restrict self-reporting to the frequency and description of behaviours rather than an assessment of their quality [51].

In this review, we used “professionalism” as our keyword in the search. However, there may be studies that measured professionalism or its dimensions without using this particular term, and they would have been omitted from our search. Another limitation is that we focused on scale development papers, and may have neglected the rigorous information available in qualitative studies.

### Conclusions

Professional behaviour is crucial in everyday practice, as pharmacists are expected to demonstrate qualities such as politeness, respect, and courtesy in dealing with patients and others. High-quality measurements of such behaviours are essential for assessing any behavioural improvement. The instruments presented to date in the literature have focused on measuring elements of professionalism among students, rather than practising pharmacists. This paper reviewed 4 important instruments developed to measure professionalism dimensions in pharmacy education. Further research is now needed on professionalism in pharmacists, and it is necessary to move from assessments in students to those now in practice. The challenge now is to develop and validate a tool for measuring pharmacists’ professional performance in the workplace. Such a tool would help in collecting standardised and measurable data for pharmacists that would be used to ensure that the activities of practising pharmacists are suitable, and to evaluate their level of performance to foster their professional growth. By doing this we can ensure improved standards of care to the community.

## Supplementary materials

Supplement 1. Audio recording of the abstract.
